# Older Adults’ Engagement in Cognitively Stimulating Activities Prior to the Pandemic Predicts Loneliness

**DOI:** 10.1093/geroni/igab046.1199

**Published:** 2021-12-17

**Authors:** Lilian Azer, Isaac Quintanilla Salinas, Esra Kürüm, Leah Ferguson, Elizabeth Davis, Weiwei Zhang, Carla Strickland-Hughes, Rachel Wu

**Affiliations:** 1 University of California, Riverside, Riverside, California, United States; 2 University of the Pacific, University of the Pacific, California, United States; 3 University Of California - Riverside, Riverside, California, United States

## Abstract

Loneliness, which may be more prevalent in older than younger adults, may lead to increased subjective cognitive decline and cognitive impairment may in turn predict perceived loneliness. COVID-19 physical distancing restrictions may exacerbate perceived loneliness, especially that experienced by older adults. The present study investigated whether self-reported cognitive abilities (i.e., executive functions) would predict loneliness during the COVID-19 pandemic. Younger (YA; n = 136, 18-35 years), middle-aged (MA; n = 126, 36-54 years), and older (OA; n = 171, 55-88 years) adults completed questionnaires assessing self-reported executive functions (EF) and perceived loneliness using the BRIEF-A and UCLA Loneliness scale respectively. Forty-nine of the 171 older participants partially completed a cognitive learning intervention, which has previously been found to increase EF. In the current study, age group did not significantly predict perceived loneliness. However, OA who participated in the prior intervention reported less loneliness than those who did not participate in the intervention. Additionally, OA who participated in the intervention and self-reported worse EF during the current study, also reported feeling lonelier than adults who did not participate in the intervention. Although results from our prior research found most OA who participated in the intervention improved their EF, the results from the current study suggest that it left them more susceptible to the negative effects of physical distancing restrictions if they had lower self-reported EF during the pandemic. Decreased engagement, real or perceived, in cognitively stimulating activities due to the pandemic strengthens the relationship between lower self-reported EF and perceived loneliness.

